# Medical Device Regulation: Should We Care About It?

**DOI:** 10.1007/s44200-022-00014-0

**Published:** 2022-03-31

**Authors:** Elisabetta Bianchini, Christopher Clemens Mayer

**Affiliations:** 1 Institute of Clinical Physiology, Italian National Research Council (CNR), Pisa, Italy; 2 Center for Health & Bioresources, Medical Signal Analysis, AIT Austrian Institute of Technology GmbH, Vienna, Austria

**Keywords:** MDR, Medical device, Regulation, Vascular ageing

## Abstract

Medical devices are subject to strict regulatory and approval processes to enter the market and to be used by operators and patients. These are needed to guarantee the users’ safety. The different activities of these processes have important implications for all involved stakeholders and for the whole lifecycle of a medical device. The aim of this work is to provide an overview of some key aspects of the new EU Medical Device Regulation and to show why researchers, innovators and clinicians should care about it. Awareness of regulatory requirements can improve the innovation process and its efficiency in terms of both social and ethical impact, but this awareness needs to be raised in the upcoming months and years. One can shortly say “yes, one needs to take care” of the new EU Medical Device Regulation. First and foremost, it is crucial for the sake of the users’ safety, which is the regulation’s intrinsic goal. Second, it should not just be seen as an obstacle for new innovations in the medical domain, but as a chance as it can provide new opportunities.

## Background

According to the European Commission [1], “medical devices have a fundamental role in saving lives by providing innovative healthcare solutions for the diagnosis, prevention, monitoring, prediction, prognosis, treatment or alleviation of disease”. More specifically, the European Union (EU) market includes thousands of medical devices. Thus, the sector is not only essential for healthcare services to citizens, but as well crucial for the global economy.

Within this context, there is a need to guarantee safety and availability of different systems, aimed at improving or saving lives. Since this is a huge and heterogeneous market, dedicated rules are needed. Recently, the new Regulation MDR (EU) 2017/745 [2] came into force introducing further and more explicit requirements related to the activities of the players involved in the development process of medical devices.

## Medical Devices Regulation

**Fig. 1:**
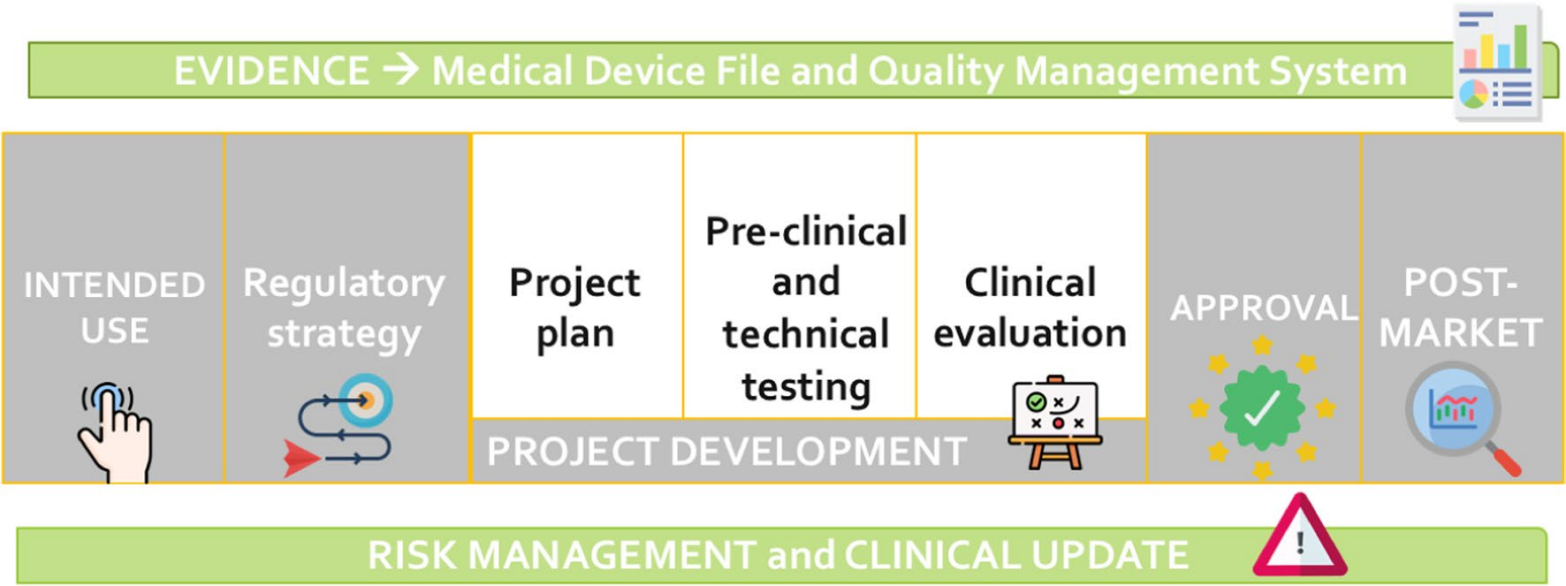
Summary of the market approval process for a medical device. After identifying the intended use of a new system, a regulatory strategy can be planned. During the project development tests are performed and data are collected. Once the evidence in terms of safety and performance of the device is adequate, approval can be obtained. Post-market activities have to be implemented for the whole life-cycle of the device taking into account benefits-risks balance and providing evidence through controlled documentation and procedures

Regulation is based on rules about the development, validation, and maintenance of medical devices. More specifically, medical devices are defined as systems intended to be used in humans for diagnosis, prevention, monitoring, treatment or alleviation of a disease or an injury [2]. The application of specific rules in this field leads to regulated products supervised by Government and Authorized Entities (i.e., Notified Bodies) for market approval as described in Fig. 1. The main aim of this process is to guarantee the introduction and maintenance of effective technologies that can be safely adopted by the users [3]. Many of the technologies developed and adopted for vascular ageing assessment are intended to be used for the monitoring and the prevention of vascular alterations and thus fall into the medical device framework [4]. Awareness of the basic concepts related to the approval to market and maintenance processes is a valuable background for the main involved stakeholders that can speed-up the innovation process in the field.

Icons’ source for the picture: https://www.flaticon.com

## The Innovation Process

**Fig. 2:**
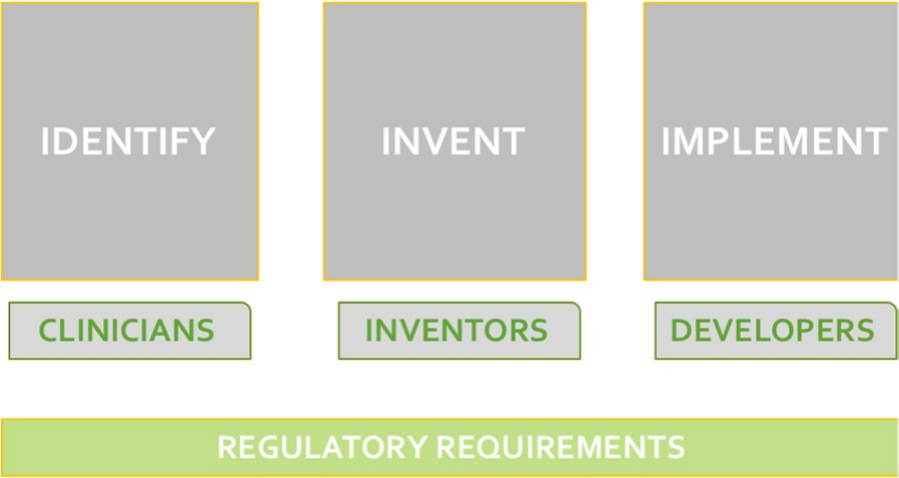
Within the Biodesign framework describing the innovation process for medical technologies [8], unmet clinical needs have to be identified, solutions to address them have to be invented and systems for the end-users have to be implemented. Different stake-holders are involved and each phase has to deal with Regulation

Innovation in medicine and its relation with legislation are often under debate due to the important interplay between on the one hand the priority on the safety for users, and on the other hand simultaneously guarantying technological improvement and evolution provided by inventors [5–7]. Models are available for the description of new medical technology development. According to the Stanford Biodesign framework, the innovation process can be managed according to the “three I” main phases [8]. This approach can be applied to different fields of life science and it is particularly suitable for cardiovascular medicine given its wide range of clinical applications [9]. In particular, the process suggest that unmet clinical needs have to be *identified*, solutions to address them have to be *invented* and systems for the end-users have to be *implemented* (Fig. 2). When detailing these steps, it clearly emerges that each of them has to deal with regulatory aspects. The identification phase requires to take into account the main stakeholders and their roles, the invention process needs to analyze legislation requirements and the implementation activity includes a regulatory strategy. Consequently, regulation is a transversal theme of medical technology development impacting the whole lifecycle of a device and it should be considered already at the design phase. This consideration and the release of a new EU Regulation pose emphasis on the key role of clinicians and scientists within the process and because of their awareness about specific requirements of a medical device.

## The New EU Regulation

### General Description

Advances and diffusion of innovative solutions in medicine have led to discussion about interpretation and application of the past Directives related to medical devices (Directives 93/42/EEC and 2007/47/EC). In this context, a new regulatory framework which governs market access to the European Union has been planned and implemented to provide legislation aligned as much as possible to technological evolution and that could be uniform throughout the EU panorama. MDR (EU) 2017/745 Medical Devices Regulation was officially published on May 5, 2017 and entered into force on May 26, 2017. The legislation provides a three-year transition time (extended by 1 year due to the COVID-19 pandemic, until May 26, 2021) to meet the requirements of the MDR and allow manufacturers to place medical devices on the EU market following new rules. In contrast to the past Directives, regulations are directly applicable into national law, thus, reducing discrepancies around the European Union. In general, no requirements from the Directives have been removed and MDR added new ones. It is worth noting that all the medical devices have to be re-certified according to the new process, introducing a life-cycle approach to safety, supported by data.

### Main Changes in Legislation Impacting the Activities of Clinicians and Scientists

MDR was created as a real new Certification with further requirements (e.g., obligation for the manufacturers of a new role, within the organization, responsible for regulatory compliance) and stricter measures (e.g., more rigorous post-market surveillance and vigilance) in line with the current global medical device market. More specifically, the introduction of new requirements aims to guarantee the safety of users thanks to greater transparency and better traceability of medical devices. Among the changes introduced by the new Regulation, some aspects are worth underlining due to their potential impact on activities performed by clinicians and scientists and will be described in the next paragraphs.

#### Classification Rules and Scope Expansion

**Fig. 3:**
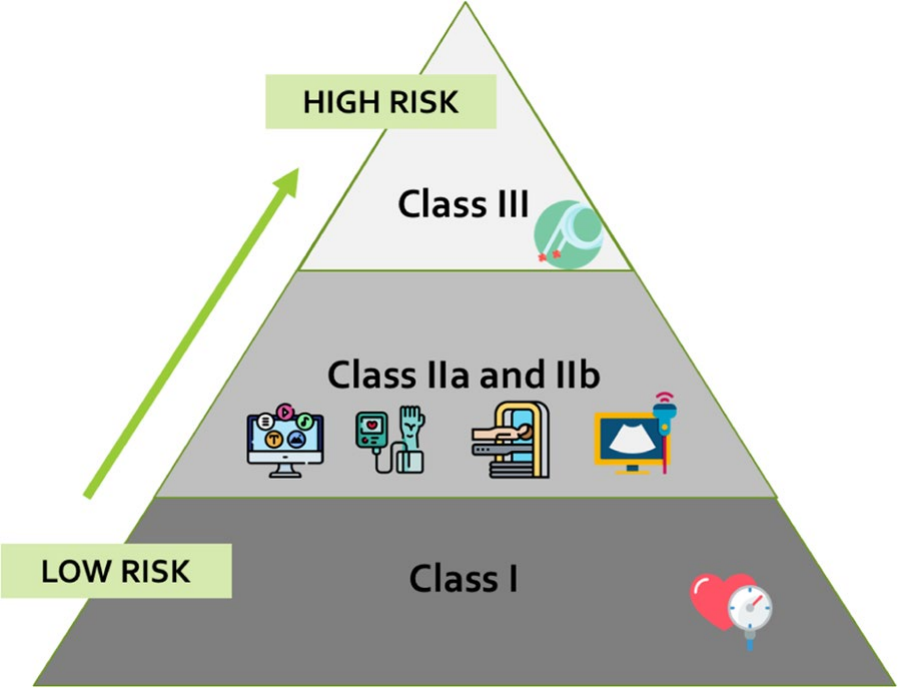
Risk based classification of medical devices according to EU Regulation. As reported in [4] many medical devices adopted for vascular ageing assessment fall at least in class IIa. Icons’ source for the picture: https://www.flaticon.com

Medical devices are classified according to a risk-based approach considering safety of users a priority and evaluating the potential risks associated with use of the device. This framework adopts criteria (e.g., invasiveness, source of energy, potential toxicity etc.), named “classification rules” (Annex VIII of MDR). These criteria lead to four main classes: I (lowest risk), IIa, IIb, III (highest risk), see Fig. 3. MDR has a wider scope and reclassifies some types of systems with respect to the previous Directives. For instance, the Regulation refers to techniques for disinfecting and sterilizing other medical devices that previously were not included. An interesting example related to innovative approaches widely adopted in medicine and in vascular ageing assessment is the higher attention placed on Medical Device Software (MDSW). Qualification has been made clearer, since “software which is intended to process, analyze, create or modify medical information” may be defined as a medical device “if the creation or modification of that information is governed by a medical intended purpose” [10]. In addition, a new classification rule (MDR rule 11, annex VIII) specifically for software has been introduced and it provides more explicit requirements than in the past. These changes will lead to a clarification for the qualification of some tools as medical devices and the update of many medical software products, already approved, to classes of higher risk and therefore to more complex certification processes. As a consequence, a more rigorous development and maintenance process for these is required. The clarification and re-classification have an impact on the activities of all involved actors starting from the design phase.

#### Explicit Obligation of a System for Risk Management

Risk has to be managed not only in the classification phase, but for the whole lifecycle of a device. MDR (Article 10, 2) requires Manufacturers to establish, document, implement and maintain a safety risk management system for the product. Risks associated with the device, including those related to human factors, must be reduced as far as possible and a process for risk management must be implemented. All residual risks must be outbalanced by the benefits related to the intended use of the device and risk mitigations must be effective throughout the life of the device. This transversal approach implies that risk-related requirements must be considered since the design phase, where inventors, clinicians and scientists might be involved. Moreover, the users’ role and their feedback are crucial and required for the whole lifecycle of the device. Finally, the value of the device introduced on the market needs to be described in terms of “clinical benefit leading to positive impact on patient-relevant clinical outcome” [2, 11]. Clearly, this is a complex process involving not only the manufacturer but the whole scientific community whose experiences and areas of expertise play a fundamental role.

#### More Rigorous Clinical Evidence About Safety and Performance

**Fig. 4:**
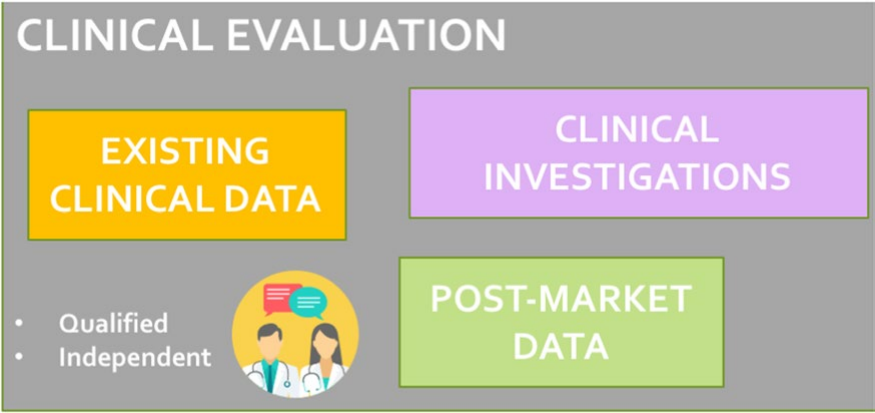
Clinical evidence can be derived from different sources: existing data or new studies. The activity needs to be implemented both for new systems and for those already on the market. Icon’s source for the picture: https://www.flaticon.com

Clinical Evaluation is strictly related to the risk management mentioned above. MDR requires a more systematic approach and underlines the importance of planning and performing this activity, based on clinical data that provide evidence about safety and performance, as well as on the acceptability of the benefit-risk in adopting it. The process is based on the collection and assessment of data relating to a specific device. The analysis can include already existing data (e.g., related to similar or equivalent devices) or new data (Fig. 4). The need for new studies in humans, named clinical investigations, depends on the ability of existing data to adequately address the benefit/risk profile, claims, and side-effects to comply with the applicable Requirements [6]. The key role of a sponsor of a clinical investigation is clearly stated in MDR Article 2(49), as: “any individual, company, institution or organization which takes responsibility for the initiation, for the management and setting up of the financing of the clinical investigation” [2]. Once a system is approved for use, clinical data have to be continuously updated through a post-market activity. It is worth noting that this complex work is also based on the contributions of several stakeholders and requires the support of independent and qualified personnel [12–14]. Moreover, evidence derived by the adoption of an independent medical device registry could be valuable for the whole process.

#### Creation of a Framework for Traceability

The new Regulation reinforces and supports the implementation of device traceability with the introduction of the UDI system (unique device identification, Art 28), a unique numeric or alphanumeric code [15]. In addition, MDR introduces Eudamed (https://ec.europa.eu/tools/eudamed/#/screen/home, Art. 33–34). It is a platform aimed to collect key information from all EU countries in a single publicly available database. This database will allow to monitor the safety and performance of medical devices available on the EU market and to improve transparency of data related to them. Eudamed includes six main modules related to: stakeholder registration, unique device identification (UDI) and device registration, notified bodies and certificates, clinical investigations and performance studies, vigilance and market surveillance.

These tools implement a model that requires efforts by the actors and provides more information to everybody than in the past. The approach is based on more rigorous post market activities and more data available for the Society, without discrepancy around EU. It aims to reinforced traceability and transparency for the medical devices market, an improvement strongly suggested and discussed, especially for high-risk systems, by the Scientific Community [16–18].

## Discussion and Conclusions

The European Union’s new regulatory framework for medical devices has introduced changes that have implications for systems already on the market and for the development of new devices.

In particular, contributions by all stakeholders, including the scientific community, are required for a complex and transversal approach based on risk management, clinical evidence, traceability and transparency. Moreover, MDR has a wider scope and introduces further classification rules with respect to the previous Directives. A specific example is Medical Device Software, that are frequently adopted in vascular ageing assessment, both in clinical and research activities. Medical Device Software plays a crucial role in the modern medicine. It was already included in the previous Directives, but has now been more clearly described both in terms of qualification and classification. Open issues regarding operative guidelines are still open especially for the most innovative implementations. As an example, the availability of large data sets together with the expansion of computational models and power is providing a huge step ahead for the role of bio-signal and image analysis in medicine thanks to the development of Artificial Intelligence (AI) approaches. AI solutions are based on dynamic and ever evolving systems and validation and approval processes, based on risk assessment, need to be adapted also to these types of applications. Strategies and procedures based on scientific approaches, which are able to support innovation and to maintain the safety for users as a priority, are desirable and need the involvement of experts [14].

As previously mentioned, within the innovation process, scientists and clinicians play a key role as inventors, developers and validators [8]. In addition, the device’s life-cycle approach supported by data, required by MDR, clarifies also the continuous need of expertise and involvement from the scientific community to guarantee safety and performance related to the adopted technologies. The current pandemic period, as described in [19], has been an opportunity to underline the role of scientists and clinicians within this process. In crisis conditions, specific questions can be rapidly addressed only by experts who have to be able to communicate answers to the society including politicians and regulators. On the one hand, regulatory frameworks open to Science and its results with a safe but also flexible approach are desirable for providing the best health strategies to patients and operators. On the other hand, an alert scientific community aware of the process and requirements for approval of new medical devices and maintenance of those already in use, can strongly improve the effectiveness of modern medicine. Osmosis among the different actors is crucial for the creation of a fruitful and solid basis supporting evolution of technology and application of rules.

The technology adoption lifecycle for disruptive innovation has been described by Moore's theories [20], where five main segments of users are recognized: innovators, early adopters, early majority, late majority and laggards. Currently, we, as community involved in the vascular ageing assessment, are probably dealing with the most difficult step: the transition between early adopters and early majority. Several initiatives are planned and implemented in this direction, including development of more usable technologies, validation and clinical studies, definition of guidelines and networking projects [21, 22]. A further piece for crossing this chasm could be built thanks to increased awareness of regulatory concepts and of the key role that our scientific community can play in implementation and debate around them.

In conclusion, one can say “yes, one needs to take care” of the new Medical Device Regulation. First and foremost, it is crucial for the sake of the users’ safety. Secondly, it should not just be seen as an obstacle for new innovations in the medical domain, but as a chance. The whole process aims to ensure quality and efficacy of new devices, and thus help companies to enter the market and to be distinguishable from non-medical or non-certified devices. Anyway, there is still uncertainty for the involved stakeholders, thus debating and raising awareness is crucial. To do so, as a first step investigation of perception and identification of the current state of knowledge and of gaps in knowledge (e.g., via a survey) are important.

## Data Availability

Not applicable.

## Abbreviations

EU: European Union
MDR (EU) 2017/745 and MDR: European Union’s Medical Device Regulation
MDSW: Medical Device Software
UDI: Unique device identification

## References

[1] Overview | Public Health n.d. https://ec.europa.eu/health/md_sector/overview_en (accessed Nov 1, 2021).

[2] REGULATION (EU) 2017/745 OF THE EUROPEAN PARLIAMENT AND OF THE COUNCIL—of 5 April 2017—on medical devices, amending Directive 2001/ 83/ EC, Regulation (EC) No 178/ 2002 and Regulation (EC) No 1223/ 2009 and repealing Council Directives 90/ 385/ EEC and 93/ 42/ EEC n.d. https://eur-lex.europa.eu/legal-content/EN/TXT/PDF/?uri=CELEX:32017R0745.

[3] Ss A (2020). The essential principles of safety and effectiveness for medical devices and the role of standards. Med Devices (Auckl).

[4] Mayer CC, Francesconi M, Grandi C, Mozos I, Tagliaferri S, Terentes-Printzios D (2021). Regulatory requirements for medical devices and vascular ageing: an overview. Heart Lung Circ.

[5] Guerra-Bretaña RM, Flórez-Rendón AL (2018). Impact of regulations on innovation in the field of medical devices. Res Biomed Eng.

[6] Melvin T, Torre M (2019). New medical device regulations: the regulator’s view. EFORT Open Rev.

[7] Lind KD. Implantable devices: regulatory framework and reform options—AARP insight on the issues 2017.

[8] Zenios SA, Makower J, Yock PG (2010). Biodesign: the process of innovating medical technologies.

[9] Schwartz JG, Kumar UN, Azagury DE, Brinton TJ, Yock PG (2016). Needs-based innovation in cardiovascular medicine: the stanford bio-design process. JACC Basic to Transl Sci.

[10] Guidance on Qualification and Classification of Software in Regulation (EU) 2017/745 – MDR and Regulation (EU) 2017/746 – IVDR - DocsRoom - European Commission 2019. https://ec.europa.eu/docsroom/documents/37581 (accessed Dec 10, 2021)

[11] Medical Device Medical Device Coordination Group Document. MDCG 2020–6 Regulation (EU) 2017/745: clinical evidence needed for medical devices previously CE marked under Directives 93/42/EEC or 90/385/EEC . n.d. https://ec.europa.eu/health/system/files/2020-09/md_mdcg_2020_6_guidance_sufficient_clinical_evidence_en_0.pdf.

[12] Fraser AG, Byrne RA, Kautzner J, Butchart EG, Szymanski P, Leggeri I (2020). Implementing the new European regulations on medical devices-clinical responsibilities for evidence-based practice: a report from the regulatory affairs committee of the European society of cardiology. Eur Heart J.

[13] Campbell B, Wilkinson J, Marlow M, Sheldon M (2018). Long-term evidence for new high-risk medical devices. Lancet.

[14] Fraser AG, Nelissen RGHH, Kjærsgaard-Andersen P, Szymański P, Melvin T, Piscoi P (2021). Improved clinical investigation and evaluation of high-risk medical devices: the rationale and objectives of CORE-MD (Coordinating Research and Evidence for Medical Devices). EFORT Open Rev.

[15] Bianchini E, Francesconi M, Testa M, Tanase M, Gemignani V (2019). Unique device identification and traceability for medical software: a major challenge for manufacturers in an ever-evolving marketplace. J Biomed Inform.

[16] Fraser AG, Butchart EG, Szymański P, Caiani EG, Crosby S, Kearney P (2018). The need for transparency of clinical evidence for medical devices in Europe. Lancet (London, England).

[17] Tanimoto T, Saito H, Sawano T, Shimada Y, Ozaki A (2019). Transparency of clinical evidence for medical devices in Europe. Lancet (London, England).

[18] Fraser AG, Butchart EG, Szymański P (2019). Transparency of clinical evidence for medical devices in Europe—authors’ reply. Lancet.

[19] Fraser AG, Szymanski P, Macintyre E, Landray M (2020). Regulating drugs, medical devices, and diagnostic tests in the European Union: early lessons from the COVID-19 pandemic?. Eur Heart J.

[20] Moore GA. Crossing the Chasm, 3rd Edition: marketing and Selling Disruptive Products to Mainstream Customers. HarperCollins; 2014 ISBN: 9780062292988, ISBN 10: 0062292986 Imprint: Harper Business On Sale: January 28, 2014.

[21] Climie RE, Mayer CC, Bruno RM, Hametner B (2020). Addressing the unmet needs of measuring vascular ageing in clinical practice-European cooperation in science and technology action vascagenet. Artery Res.

[22] Mayer CC, Climie RE, Hametner B, Bruno RM (2020). The European COST action VascAgeNet fostering innovation—when industry comes to science. Artery Res.

